# Crystal Structure Of Photorespiratory Alanine:Glyoxylate Aminotransferase 1 (AGT1) From *Arabidopsis thaliana*

**DOI:** 10.3389/fpls.2019.01229

**Published:** 2019-10-11

**Authors:** Aaron H. Liepman, J. Vijayalakshmi, Daniel Peisach, Brian Hulsebus, Laura J. Olsen, Mark A. Saper

**Affiliations:** ^1^Biology Department, Eastern Michigan University, Ypsilanti, MI, United States; ^2^Department of Biological Chemistry and LSA Biophysics Program, University of Michigan, Ann Arbor, MI, United States; ^3^Department of Molecular, Cellular, and Developmental Biology, University of Michigan, Ann Arbor, MI, United States

**Keywords:** dimer, tetramer, substrate specificity, PLP, *sat* mutant, serine:glyoxylate aminotransferase, photorespiration

## Abstract

Photorespiration is an energetically costly metabolic pathway for the recycling of phosphoglycolate produced by the oxygenase activity of ribulose-1,5-bisphosphate carboxylase/oxygenase (RUBISCO) to phosphoglycerate. *Arabidopsis* alanine:glyoxylate aminotransferase 1 (AGT1) is a peroxisomal aminotransferase with a central role in photorespiration. This enzyme catalyzes various aminotransferase reactions, including serine:glyoxylate, alanine:glyoxylate, and asparagine:glyoxylate transaminations. To better understand structural features that govern the specificity of this enzyme, its crystal structures in the native form (2.2-Å resolution) and in the presence of l-serine (2.1-Å resolution) were solved. The structures confirm that this enzyme is dimeric, in agreement with studies of the active enzyme in solution. In the crystal, another dimer related by noncrystallographic symmetry makes close interactions to form a tetramer mediated in part by an extra carboxyl-terminal helix conserved in plant homologs of AGT1. Pyridoxal 5′-phosphate (PLP) is bound at the active site but is not held in place by covalent interactions. Residues Tyr35′ and Arg36′, entering the active site from the other subunits in the dimer, mediate interactions between AGT and l-serine when used as a substrate. In comparison, AGT1 from humans and AGT1 from *Anabaena* lack these two residues and instead position a tyrosine ring into the binding site, which accounts for their preference for l-alanine instead of l-serine. The structure also rationalizes the phenotype of the *sat* mutant, Pro251 to Leu, which likely affects the dimer interface near the catalytic site. This structural model of AGT1 provides valuable new information about this protein that may enable improvements to the efficiency of photorespiration.

## Introduction

The enzyme ribulose-1,5-bisphosphate carboxylase/oxygenase (RUBISCO) catalyzes most carbon fixation by photosynthetic organisms (Peterhansel et al., 2010). In current atmospheric conditions, it is estimated that the ratio of carboxylation:oxygenation of ribulose-1,5-bisphosphate (RuBP) by RUBISCO is approximately 3:1 (Sharkey, 1988); however, oxygenation becomes more pronounced at higher temperatures and in dry conditions (Peterhansel et al., 2010). When RUBISCO catalyzes carboxylation of RuBP, two molecules of 3-phosphoglycerate (PGA) are formed, and these PGA molecules enter the Calvin cycle to form sugars. When RUBISCO instead oxygenates RuBP, one molecule of PGA and one molecule of 2-phosphoglycolate are formed. This phosphoglycolate cannot directly enter the Calvin cycle and instead is converted to PGA *via* a coordinated metabolic pathway called photorespiration, which takes place in chloroplasts, peroxisomes, and mitochondria. The photorespiratory pathway involves the actions of numerous enzymes and metabolite transporters and results in the release of CO_2_ and ammonia and concomitant consumption of adenosine triphosphate (ATP) and reducing equivalents (Peterhansel et al., 2010).

Two different types of peroxisomal aminotransferases are active within the photorespiratory pathway, glutamate:glyoxylate aminotransferase (GGAT) and serine:glyoxylate aminotransferase (SGAT) (Igarashi et al., 2003; Liepman and Olsen, 2003). In *Arabidopsis thaliana*, two genes encode enzymes with GGAT activity, At1G23310 (GGT1) and At1G70580 (GGT2). These enzymes are members of aminotransferase class I, and they each catalyze transamination reactions using l-glutamate or l-alanine as amino donor and glyoxylate or pyruvate as amino acceptor (Liepman and Olsen, 2003). Plants harboring a mutation in the *GGT1* gene exhibited a conditional photorespiratory phenotype of stunted growth when cultured in normal CO_2_ (0.03%); this phenotype was not evident when plants were grown in elevated CO_2_ (0.3%) (Igarashi et al., 2003). The *alanine:glyoxylate aminotransferase 1* (*AGT1*) gene (At2G13360) of *Arabidopsis* encodes a multifunctional class IV aminotransferase protein that catalyzes transamination reactions using l-serine, l-alanine, and l-asparagine as amino donors and glyoxylate, pyruvate, and hydroxypyruvate as amino acceptors (Liepman and Olsen, 2001; Kendziorek and Paszkowski, 2008; Zhang et al., 2013). A point mutation in the AGT1 protein (AGT1-P251L) renders *sat* mutant plants lethally stunted when grown in normal atmospheric conditions (Somerville and Ogren, 1980; Liepman and Olsen, 2001). Outside of photorespiration, AGT1 also has been implicated in the asparagine catabolic pathway (Ivanov et al., 2012), pathogen resistance (Taler et al., 2004), and salt tolerance (Yang et al., 2013).

Because an estimated 25% of carbon fixed during photosynthesis is lost *via* photorespiration, this pathway is an appealing target for engineering efforts aiming to boost efficiency. Various approaches that may lead to such improvements are being explored, including altering the sequence of RUBISCO to improve its carboxylation rate (Whitney et al., 2011), the introduction of carbon concentration mechanisms (Lin et al., 2014a; Lin et al., 2014b), and implementation of various photorespiratory metabolic bypass pathways (Kebeish et al., 2007; Carvalho et al., 2011; Maier et al., 2012). A recent field experiment of tobacco plants engineered with a combination of bypass pathways resulted in an approximately 40% increase of biomass in transgenic lines (South et al., 2019). While these studies show great promise, it is likely that a multifaceted approach involving a combination of strategies will be needed to fine-tune photosynthesis and photorespiration for maximal efficiency and yield benefit. To inform these efforts, detailed knowledge about the structures and functions of photorespiratory proteins will be needed. Here we describe the structure of AGT1 from *Arabidopsis thaliana*, solved using X-ray crystallography at a resolution of 2.1 Å, and highlight the molecular determinants of its substrate specificity.

## Results And Discussion

Full-length alanine-glyoxylate aminotransferase (AGT1) from *A. thaliana* was expressed in *Escherichia coli* (Liepman and Olsen, 2001) and purified to near homogeneity using ion exchange and gel filtration chromatography. Native crystals were grown in the presence of the cofactor pyridoxal 5′-phosphate (PLP), and the structure was phased to a resolution of 3.0 Å by multiple isomorphous replacement with four heavy atom derivatives using *SOLVE* and *RESOLVE* (final figure of merit = 0.66). The structure, comprised of two AGT1 molecules (chain A: residues 3–401, chain B: residues 2–401) related by noncrystallographic symmetry, two PLPs, one formate, one chloride ion, and 342 water molecules, was refined to 2.2 Å with *PHENIX* (Adams et al., 2010) (crystallographic *R*_work_ = 0.139 and *R*_free_ = 0.170; details in **Table 1**). Data were also obtained from an AGT1 crystal grown in the presence of 20 mM l-serine. This isomorphous structure contained two AGT1 molecules (residues 3–401 in each), two PLPs, one hydroxypyruvate, and 422 water molecules. This structure was refined to 2.1 Å with *R*_work_ = 0.115 and *R*_free_ = 0.166. Molecules from each crystal form were almost identical; root mean square distance between α-carbon atoms was 0.10 Å for 798 α-carbons. The results described herein are for the AGT1 crystals grown in the presence of l-serine and are valid for both structures unless explicitly mentioned.

**Table 1:** Crystallographic data and refinement statistics for alanine:glyoxylate aminotransferase 1 (AGT1) crystals.

Crystal	AGT1 native PDB ID 6PK3	AGT with 20 mM Ser PDB ID 6PK1
Wavelength (Å)	1.54	1.54
Resolution range (Å)	22.6–2.18 (2.26–2.18)	22.16–2.10 (2.18–2.10)
Space group	*P*2_1_2_1_2	*P*2_1_2_1_2
Unit cell (Å)	*a* = 139.18, *b* = 62.29, *c* = 97.01	*a* = 139.18, *b* = 62.38, *c* = 96.98
No. molecules/a.u.	2	2
Total measurements	131,288	236,629
Unique reflections	39,728 (2,057)	45,371 (2,622)
Multiplicity	3.7 (1.8)	5.0 (2.5)
Completeness (%)	89.1 (46.8)	90.6 (53.0)
Mean *I*/σ(*I*)	15.4 (3.5)	16.0 (3.8)
Wilson B-factor	23.6	19.62
R-merge	0.077 (0.209)	0.10 (0.25)
R-meas	n.a.*	0.11 (0.30)
R-pim	n.a.*	0.04 (0.16)
CC1/2	n.a.*	0.99 (0.93)
Reflections used in refinement	39,723 (2,058)	45,359 (2,624)
Reflections used for R-free	2,789 (140)	3,184 (172)
*R*_cryst_	0.139 (0.183)	0.115 (0.162)
*R*_free_	0.170 (0.202)	0.166 (0.225)
Number of nonhydrogen atoms	6,650	6,685
Macromolecules	6,272	6,224
Ligands	36	39
Solvent	342	422
Protein residues	799	801
RMS (bonds)	0.003	0.015
RMS (angles)	0.55	1.13
Ramachandran favored (%)	97.4	96.6
Ramachandran allowed (%)	2.6	3.4
Ramachandran outliers (%)	0.0	0.0
Rotamer outliers (%)	0.59	0.74
Clashscore	1.66	1.59
Average B-factor	28.2	26.0
Macromolecules	27.7	25.4
Ligands	37.9	24.1
Solvent	36.0	33.8
Number of TLS groups	11	15

*These statistics were unavailable in the version of SCALEPACK used to process the data.

Statistics for the highest-resolution shell are shown in parentheses.

Data for both crystals were collected at room temperature.

**Figure 1:**
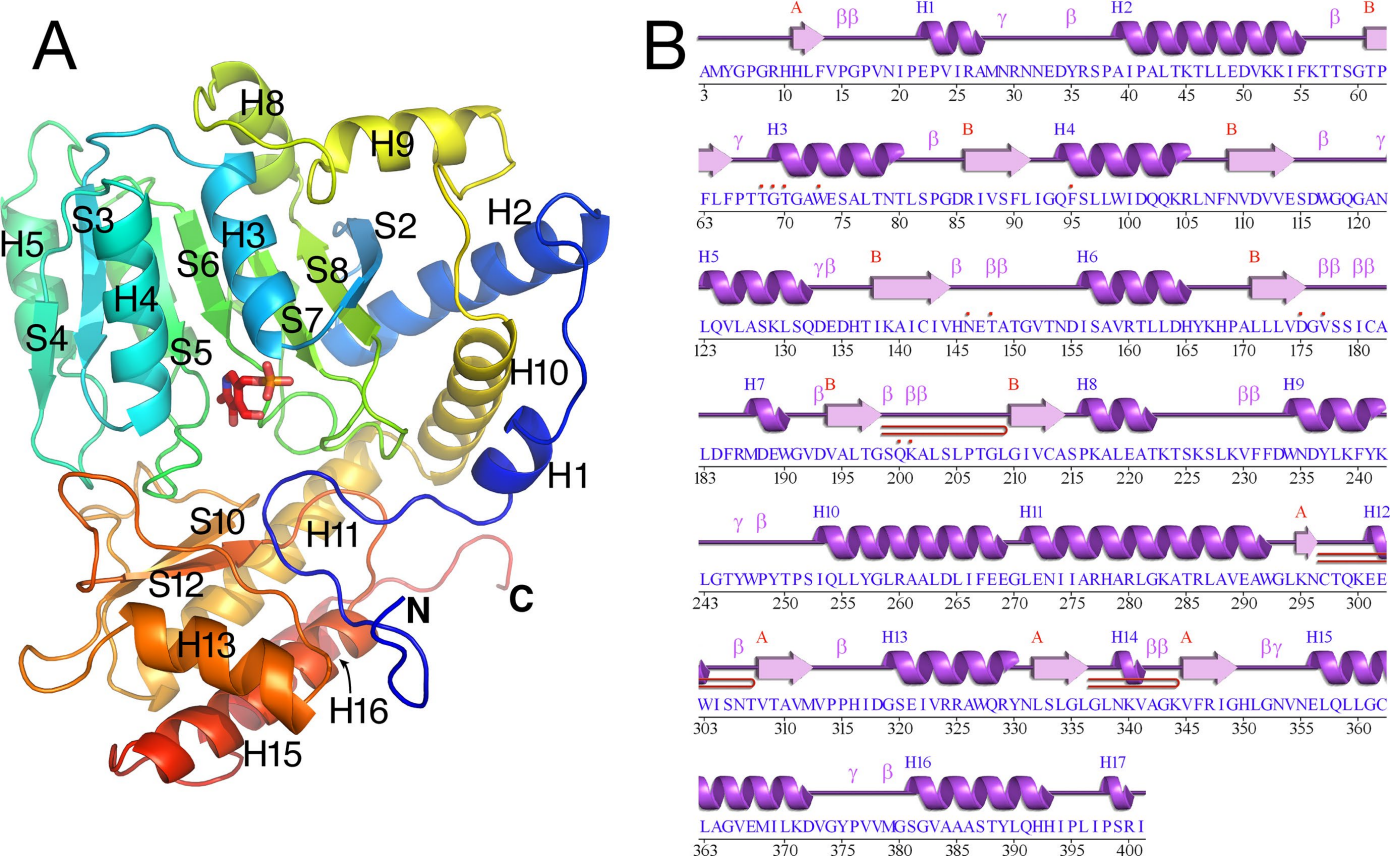
Tertiary structure of the *Arabidopsis* alanine:glyoxylate aminotransferase 1 (AGT1) monomer. **(A)** Cartoon depiction colored from blue at the N-terminus to red at the C-terminus. Coils represent helices (labeled H*n*); arrows represent β-strands (labeled S*n*). The following secondary structure elements are hidden or not shown in the diagram: S1, S9, S11, H6, H7, H12, H14, and H17. **(B)** AGT1 primary sequence annotated with corresponding helices (H*n*) and β-sheets (red letters A and B) generated by *PDBsum* (Laskowski et al., 2018). Turns are denoted by γ and β. Red dots indicate residues contacting the pyridoxal 5′-phosphate (PLP) ligand. Hairpin turns are labeled with red lines.

**Figure 2:**
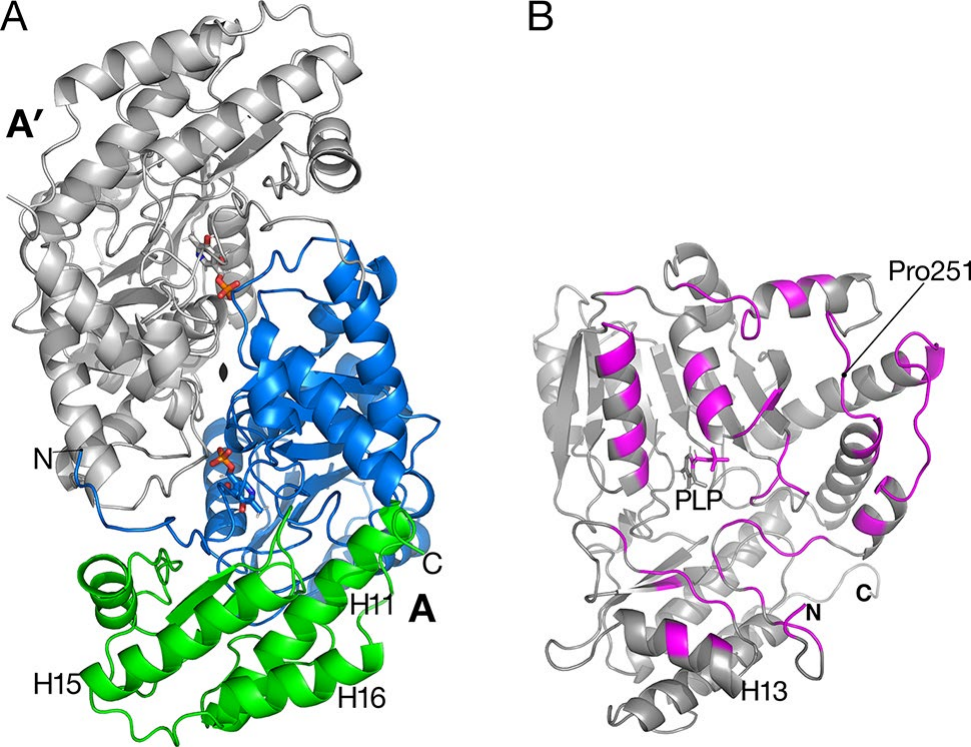
Catalytic dimer structure. **(A)** Cartoon representation of subunit A with large amino-terminal domain (residues 3–269) colored blue and smaller domain (residues 270–401) colored green. The other subunit A′ of the dimer (shown in gray) is related to subunit A by a two-fold crystallographic axis along *z*. Helices H15 and H16 are involved in the tetramer interactions described in **Figure 3**. **(B)** View of subunit A showing polypeptides (magenta) that are buried in the interface between A and A′. Residue Pro251 is the residue replaced by Leu in the *sat* mutant (**Figure 6**).

The tertiary structure of *Arabidopsis* AGT1 is characteristic of the subgroup IV class of PLP-containing enzymes (Mehta et al., 1993). This group is also classified as the class V aminotransferase family PF00266 by Pfam (Bateman et al., 2004). Each AGT1 subunit (**Figure 1A**) is comprised of a large amino-terminal domain (residues 3–269, blue in **Figure 2A**) containing a central 7-stranded mixed β-sheet with 10 helices (H1–H10) and a smaller carboxyl-terminal domain (270–401, green in **Figure 2A**) with 4 major helices (H11, H13, H15, and H16). A diagram showing the secondary structure aligned with the sequence (calculated by PDBsum; Laskowski et al., 2018) is presented in **Figure 1B**. **Table 2** lists protein structures most similar to *Arabidopsis* AGT1 from a complete search (May 2019) of the Protein Data Bank (PDB; Berman et al., 2000) calculated by the Dali server (Holm and Laakso, 2016).

**Table 2:** Structural homologs of *Arabidopsis* alanine:glyoxylate aminotransferase 1 (AGT1).

Enzyme name	Species	PDB code	% sequence identity	r.m.s.d. Å (Cα’s aligned)
Serine:pyruvate aminotransferase	*Sulfolobus solfataricus*	3ZRQ	31	1.7 (376)
Pyridoxamine-pyruvate aminotransferase	*Mesorhizobium japonicum*	2Z9X	26	1.9 (385)
Ureidoglycine-glyoxylate aminotransferase	*Bacillus subtilis*	3ISL	25	1.9 (381)
Ph1308 protein, serine aminotransferase	*Pyrococcus horikoshii*	2DR1	28	1.8 (365)
Alanine:glyoxylate aminotransferase	*Saccharomyces cerevisiae*	2BKW	25	1.7 (358)
Alanine:glyoxylate aminotransferase	*Anabaena sp.*	1VJO	29	1.8 (360)
Alanine:glyoxylate aminotransferase	*Homo sapiens*	5LUC	27	1.9 (365)
3-Hydroxykynurenine transaminase	*Anopheles gambiae*	2CH2	25	2.0 (367)
Aminotransferase	*Eubacterium rectale*	3F0H	19	1.9 (360)
Alanine:glyoxylate aminotransferase	*Mus musculus*	3KGW	25	1.9 (364)

Calculated by Dali server (http://ekhidna2.biocenter.helsinki.fi/dali) from all PDB entries (Holm and Laakso, 2016). Redundant results were omitted.

### Catalytic Dimer

A prior study of *Arabidopsis* AGT1 demonstrated that the active enzyme exists as a dimer (Liepman and Olsen, 2001). Dimers were also characteristic of crystallized AGT1 (**Figure 2A**) and consisted of an AGT1 monomer (chain A) forming a close interface with another molecule (A′) related by a crystallographic dyad along *z*. Similarly, the other molecule in the asymmetric unit, chain B, forms a catalytic dimer with chain B′ related by crystallographic symmetry. Each dimer interaction buries 2,270 Å^2^ of solvent-accessible surface area from each subunit (highlighted in magenta in **Figure 2B**), including interactions with the PLP cofactor in the respective active sites. The dimer interaction is likely essential for activity because interactions from each protomer of the dimer are needed for binding of PLP and substrate (see below).

**Figure 3:**
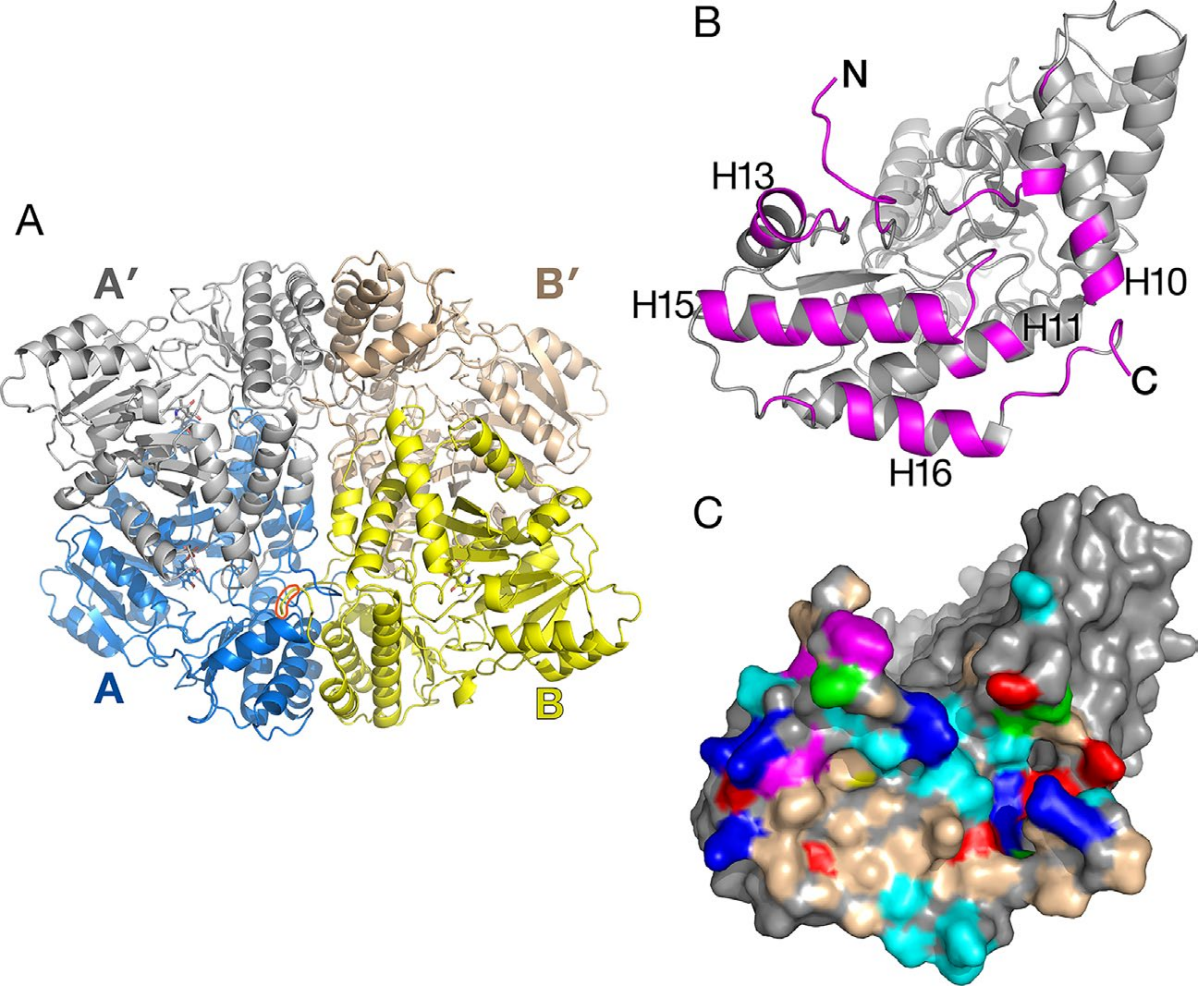
Alanine:glyoxylate aminotransferase 1 (AGT1) tetramer present in the crystal. **(A)** Shown are AGT1 molecules A and B related by a noncrystallographic two-fold axis. Each forms a catalytic dimer with A′ and B′, respectively. The orange shape outlines the C-terminal peroxisomal targeting sequence (PTS) of molecule B where it interacts with molecule A. **(B)** Molecule A is rotated about 90° to show the surface that forms the interface with molecule B. Magenta-colored polypeptides are buried in the interface. **(C)** Surface representation of molecule A [same orientation as **(B)**]. Colored surface represents residues that form the interface with molecule B. Nonpolar regions (wheat-colored) and aromatic residues (magenta-colored) contribute to the hydrophobic interactions in the tetramer interface. Other residue colors: blue, positively charged; red, negatively charged; cyan, polar; green, proline.

The human homolog of AGT1 (HsAGT1, PDB ID 1H0C; Zhang et al., 2003) and an AGT1 from *Anabaena* sp. (PDB ID 1VJO; 25% sequence identity to AGT1; Han et al., 2005) differ in several ways from *Arabidopsis* AGT1. HsAGT1 and *Anabaena* AGT have a long amino-terminal segment (∼20 residues long) that wraps around the other subunit in an extended conformation, while this feature is absent in *Arabidopsis* AGT1. In *Arabidopsis* AGT1, the amino-terminus packs near the amino-terminal end of helix H2 from the other subunit (**Figure 2A**), and the side chain of Met4 packs against Trp327 of the same subunit. Residues 5–9 loop away from the dimer surfaces.

Unlike HsAGT1 or *Anabaena* AGT1, the *Arabidopsis* AGT1 carboxyl terminus continues with an additional turn and helix (H16, residues 374–393) that packs alongside and antiparallel to the penultimate helix (H15, 356–372) and helix H11 of the small domain (residues 271–293; **Figure 2A**). Helix H16 makes important noncrystallographic symmetry interactions as described in the next section. From sequence alignment of AGT1 sequence homologs, it appears that this C-terminal helix is present only among other plant AGT1 homologs, including those from melon (accession number AAL47679), lily (AAB95218), and rice (XP_015650532), and several bacterial homologs with sequence identities greater than 30% to AGT1 (e.g., *Methylococcus* sp. [WP_010960683], *Bradyrhizobium* sp. [NP_772679], and *Thermotoga* sp. [NP_229201]).

### Noncrystallographic Tetramer

The crystallographic asymmetric unit of AGT1 contains two monomers (each from different catalytic dimers) related by a noncrystallographic two-fold axis of symmetry (NCS), which lies in the *xy* plane, 5° from the *y*-axis (molecules A and B in **Figure 3A**). Consequently, one catalytic dimer (A:A′) makes close contacts with another dimer (B:B′) related by NCS, in effect forming an apparent “dimer of dimers” (tetramer) in the crystal (**Figure 3A**). Residues buried in the interface are primarily from the amino terminus, helices H10, H13, H15, H16, and the peroxisomal targeting sequence (PTS1) at the C-terminus (392–401) (magenta in **Figure 3B**). As shown in **Figure 3C**, the surface of the AGT1 monomer involved in the NCS interface contains nonpolar and aromatic regions (wheat and magenta surfaces, respectively) that extensively contact similar regions on the NCS-related molecule. In addition, there are ∼30 hydrogen bonds between molecules A and B. Each subunit of the catalytic dimer has one of these interfaces with its noncrystallographic symmetry-related mate (A:B as well as A′:B′). In total, about 5,200 Å^2^ of the catalytic dimer solvent-accessible surface are buried in the apparent tetramer, of which about 40% is nonpolar. Analysis of the tetramer interface using PISA (Krissinel and Henrick, 2007) resulted in a complex formation significance score of 0.85, which implies that this interface is essential for tetramer complex formation.

The apparent *M*_r_ of 92,000, determined using size exclusion chromatography, was consistent with AGT1 being a homodimer (Liepman and Olsen, 2001). Although recombinant AGT1 is catalytically active as a dimer, it is possible that within the peroxisome, as in the crystal, it exists as a tetramer. The residues involved in the NCS interactions are conserved among putative plant orthologs of AGT1 and also some of the bacterial enzymes proposed to be serine:glyoxylate aminotransferases. Helix H16, which is not present in human, yeast (PDB ID 2BKW), or *Anabaena* AGT1, is an important component of the NCS interaction. Alternatively, these helices in AGT1 may mediate protein–protein interactions with other photorespiratory enzymes within peroxisomes, as it has been suggested that photorespiratory enzymes form complexes within the matrix of the peroxisome to efficiently channel photorespiratory metabolites and intermediates (Reumann, 2000).

### Catalytic Site

**Figure 4:**
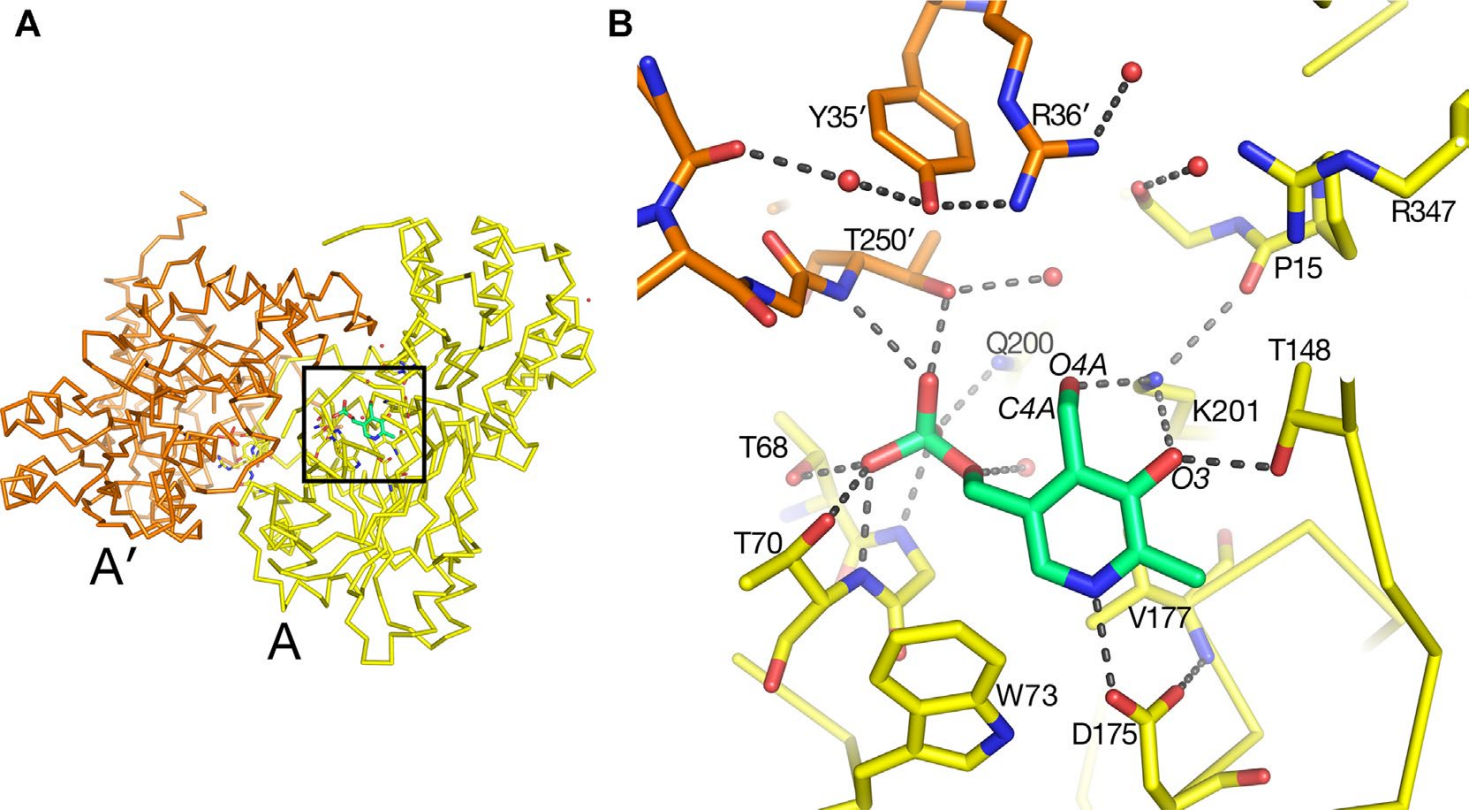
Pyridoxal 5′-phosphate (PLP)-binding site in alanine:glyoxylate aminotransferase 1 (AGT1). **(A)** Entire AGT1 dimer oriented as in the blow-up in **(B)**. Box outlines the portion of the structure depicted in **(B)**. **(B)** Yellow carbon sticks are from molecule A; orange carbon sticks are from the other subunit in the dimer (A′); green carbon and phosphate sticks are the PLP molecule bound to subunit A. Small red spheres represent water molecules. Residues are labeled with their one-letter code.

The substrate-binding site for each of the two molecules in the asymmetric unit in the native AGT1 crystal was initially refined with a PLP cofactor covalently bound to the ε-amino group (NZ) of Lys201, forming the expected internal aldimine structure common to most aminotransferases. Although continuous density for the NZ–C4A bond was initially present, large difference electron density suggested that the structural refinement process had artificially pulled the PLP molecule toward the Lys201 side chain and away from its correct position. Refinement of AGT1 with PLP in the aldehyde form, and without the internal aldimine bond to Lys201, resulted in a structure with little difference electron density and good geometry (**Figure 4**). The ε-amino group of Lys201 is within hydrogen bonding distance of O3 and O4A of PLP and the carbonyl of Pro15. A similar configuration, where no aldimine bond with the catalytic Lys was present, was observed in the AGT1 structure from *Anabaena* (Han et al., 2005). In *Arabidopsis* AGT1, the phosphate oxygens of PLP hydrogen bond to the side chains of Thr250′ (prime notation refers to the residue from the other subunit of the catalytic dimer), Gln200, Thr68 and Thr70, the amide N of Gly69 and Thr70, and water (**Figure 4B**). The PLP ring makes nonpolar contacts with Val177 on the rear (*si*) face and Phe95 on the front (*re*) face (not shown in **Figure 4B**) and Trp73 at the bottom. The conserved Asp175 forms a hydrogen bond to the PLP ring N1 at the bottom of the ring and Thr148 forms a hydrogen bond to O1 of the PLP.

In the AGT1 structure crystallized in the presence of l-serine, a pyridoxamine phosphate (PMP) molecule and no internal aldimine bond were expected, but the geometry and close distance of the PMP amino group (N4A) to the Lys201 NZ suggested that the cofactor should best be modeled as PLP.

**Figure 5:**
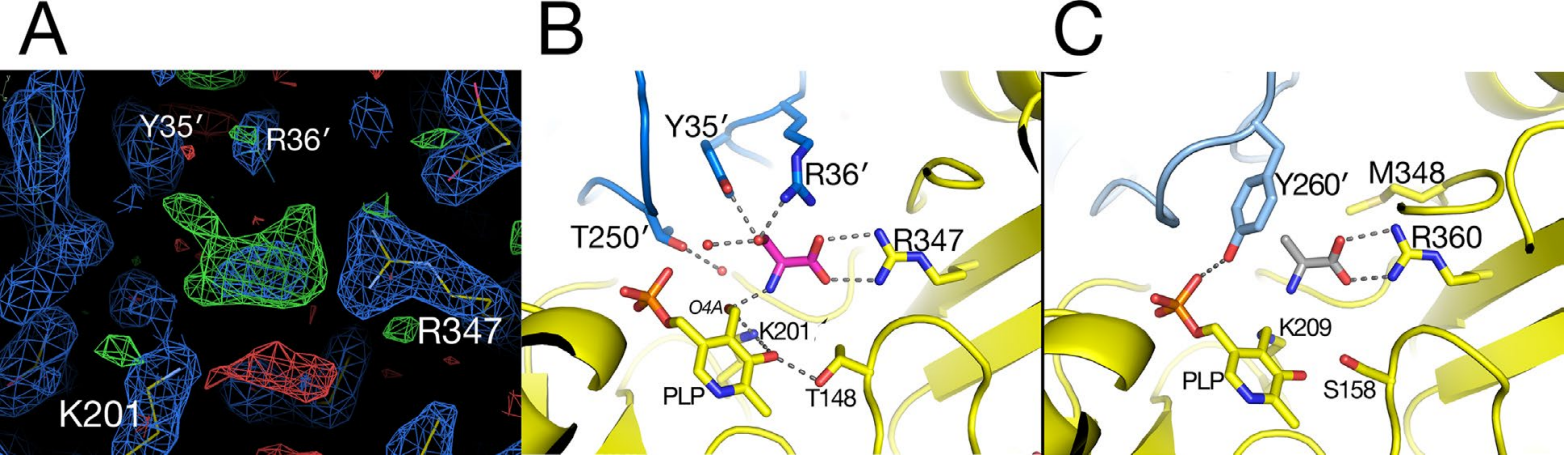
Substrate-binding site in alanine:glyoxylate aminotransferase 1 (AGT1). **(A)** Electron density near Arg347 suggestive of a bound substrate. Blue contours are from a 2*mF*_obs_–*DF*_calc_ electron density map contoured in *Coot* at 1.2 root mean square deviations above the mean. Green contours are a *mF*_obs_–*DF*_calc_ difference electron density map contoured in *Coot* at 2.25 times the root mean square deviation above the mean. Hydroxypyruvate was modeled into the electron density and refined to an occupancy of 0.67, yet no density was observed for the hydroxymethyl group (see **Supplementary Figure 1**). **(B)** l-Serine molecule (magenta) was modeled in AGT1 guided by the difference density in **(A)**. Residues with yellow carbons are from subunit A, and those with blue carbons are from the dimer’s other subunit (A′). The l-serine hydroxyl group is within hydrogen-bonding distance of Tyr35′, Arg36′, and a water molecule. **(C)** Substrate binding site of human AGT1 (PDB ID 1H0C) viewed in the same orientation as **(B)** but with an l-alanine (gray carbons) modeled in place of serine. The Tyr260′ aromatic ring may sterically clash with the hydroxyl of l-serine.

In the substrate binding site of the AGT1 crystal grown in the presence of l-serine, there was positive difference electron density suggestive of a bound molecule near the guanidinium group of Arg347 in chain A (**Figure 5A**). This density, in analogy with known structures of aminotransferase:substrate complexes, is directly above the PLP and is a likely location for the l-serine substrate or hydroxypyruvate product (ligand 3PY). The latter could be refined at partial occupancy, but there was no electron density for the hydroxyl group in the resulting map (**Supplementary Figure 1**). Using this ligand as a guide, we modeled an l-serine in the same position (**Figure 5B**). Surrounding the putative ligand (in clockwise order) are Thr250′, Tyr35′, Arg36′, Gly16, Pro15, Arg347, and Thr148. At the bottom of the site are PLP and Lys201. The side chain hydroxyl of the modeled l-serine is within hydrogen bonding distance of both Tyr35′ and Arg36′ from the other subunit of the dimer. Arg347 is found in all aminotransferases and forms ionic hydrogen bonds to the carboxylate of the modeled serine. The l-serine amino group is oriented toward the ε-amino group of the catalytic Lys201 and the aldehyde O4A of PLP. In the structure of native *Arabidopsis* AGT1, smaller difference electron density near Arg347 was modeled in chain A as a formate molecule, which was present in high concentration during crystallization, or as a chloride ion in chain B.

When the active site region of the refined structure of *Arabidopsis* AGT1 is compared to the human AGT1 (HsAGT1) structure (which prefers l-alanine as substrate), the most significant differences are in the region of *Arabidopsis* AGT1 residues Tyr35′ and Arg36′ (**Figure 5B**). In HsAGT1, the structurally equivalent residues are Ser48′ and Met49′, but these are too distant from the active site to interact with substrate. Instead, Tyr260′ of HsAGT1 enters the substrate binding site and the aromatic ring forms a nonpolar wall; the Tyr hydroxyl group hydrogen bonds to the phosphate and is not available for substrate interaction (**Figure 5C**). Lower catalytic activity of HsAGT1 with the substrate l-serine (Okuno et al., 1980) could be due to steric interactions of the serine hydroxyl with the Tyr260′ ring. The AGT1 homolog from *Anabaena* has an active site structure similar to that in HsAGT1 and also contains a conserved tyrosine, suggesting that it also may prefer l-alanine over l-serine as an amino donor. Although the enzyme from *Sulfolobus solfataricus*, with 31% sequence identity and structural similarity to *Arabidopsis* AGT1, is named a serine:pyruvate aminotransferase (PDB ID 3ZRQ), phenylalanine and other hydrophobic amino donors are actually much better substrates than serine (Sayer et al., 2012). This is consistent with the configuration of its active site, which is more similar to HsAGT1 than to *Arabidopsis* AGT1.

In *E. coli* phosphoserine aminotransferase (PDB ID 1BJO with 13% sequence identity to AGT1), another group IV aminotransferase, residues His41′ and Arg42′ occupy similar positions as Tyr35′ and Arg36′ of *Arabidopsis* AGT1 and interact with the carboxylate side chain of a substrate analog (Hester et al., 1999). Together, these results suggest that Tyr35′ and Arg36′ may enable *Arabidopsis* AGT1 to use l-serine as an amino donor substrate. Indeed, these two residues are highly conserved among a wide range of known and putative plant serine:glyoxylate amino transferases. Although not modeled here, a report indicating that l-asparagine also acts as an amino donor substrate of *Arabidopsis* AGT1 (Ivanov et al., 2012) could also be consistent with the *Arabidopsis* AGT1 active site configuration shown in **Figure 5B**.

### The *sat* Mutant Is at the Dimer Interface

**Figure 6:**
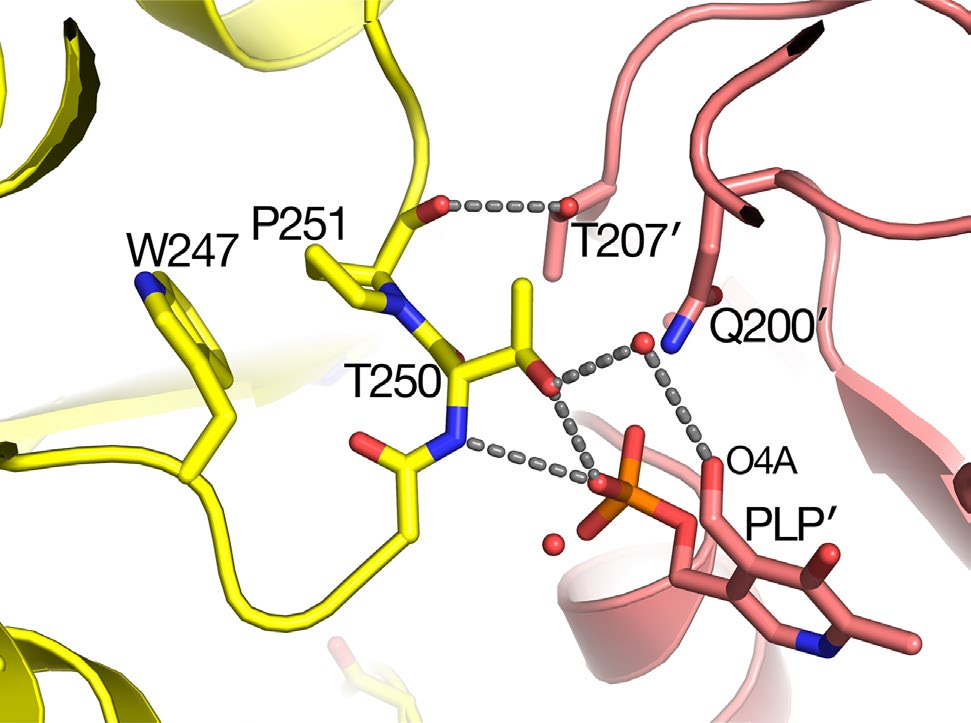
Substitution of Pro251 by Leu in the *sat* mutant results in an inactive enzyme. Carbon atoms of molecule A are colored yellow, while rose-colored atoms are from the other subunit (A′) in the catalytic dimer. Pro251 and the adjacent Thr250 make interactions with the other subunit including pyridoxal 5′-phosphate PLP′). It is likely that the Leu substitution at residue 251 would distort the backbone conformation of 250–251 because it would sterically clash with the nearby Trp247.

The *sat* mutant of *Arabidopsis thaliana* exhibits a photorespiratory air-sensitive phenotype (Somerville and Ogren, 1980) due to a point mutation that causes a substitution of Leu for Pro at position 251 of *Arabidopsis* AGT1 (Liepman and Olsen, 2001). The structure provides additional insights about the mechanistic consequences of the P251L mutation in *Arabidopsis* AGT1. Pro251 is found on an extended loop just prior to helix H10 of the larger domain (**Figure 2B**). This loop is at the dimer interface and close to the active site and PLP′ of the other subunit (**Figure 6**). The Pro251 O hydrogen bonds to the Thr207′ hydroxyl. The adjacent side chain of Thr250 also forms hydrogen bonds to the nearby PLP′ phosphate and to an active site water molecule, which hydrogen bonds to the PLP′ aldehyde (O4A). Because the Pro251 side chain packs against the ring of Trp247, replacement with leucine would likely distort the position of the loop and disrupt the hydrogen-bonds to the waters and phosphate of the nearby active site. It may also affect dimerization, rendering the enzyme inactive.

### Peroxisomal Targeting Sequence Resolved

**Figure 7:**
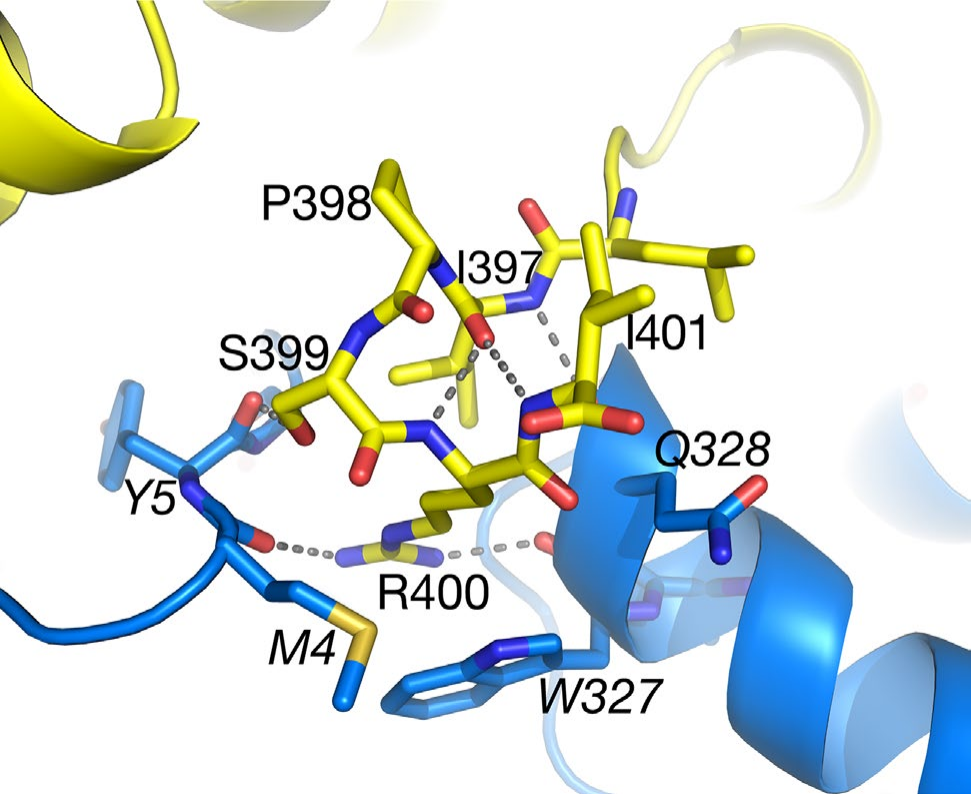
The C-terminal peroxisomal targeting sequence (PTS) sequence (Ser399–Ile401) of subunit A (yellow carbons) lies on the surface of the tetramer at the interface with subunit B (blue carbons). Also see **Figure 3A**. Ile401 is totally accessible to solvent. Italicized labels denote residues from subunit B.

Within the structure of *Arabidopsis* AGT1, the carboxyl-terminal tripeptide Ser-Arg-Ile (residues 399–401) constituting a type 1 peroxisomal targeting sequence (PTS1; Chowdhary et al., 2012) was well resolved in the electron density. In the context of the monomer or catalytic dimer, the PTS1 residues are exposed to solvent as one would expect for the *in vivo* recognition by the peroxisomal protein import machinery. However, in the tetramer interface (**Figure 7**), the side chains of residues 399–400 are on the surface but make interactions with the NCS-related chain B (carbons in blue). The PTS1 forms one turn of an α-helix facilitated by hydrogen bonds from the carbonyl of Ile397. The Ser399 hydroxyl forms a hydrogen bond to the carbonyl of residue 5 from the NCS-related molecule (chain B) and a water in the interface. Arg400 packs between residues 4 and 5 and residues 327–329 of chain B and forms hydrogen bonds to chain B residues Met4 O, Trp327 O, and Asn331 OD1. Only Ile401 is fully solvent exposed. These interactions would make it unlikely for the tetramer to exist in the cytoplasm where the PTS1 must be recognized by its receptor. In contrast, the PTS1 in the HsAGT1 dimer structure is also solvent exposed but not resolved in the electron density map. When HsAGT1 is in complex with its peroxisomal receptor Pex5p, the PTS1 sequence (Lys-Lys-Leu) exists in an extended conformation with extensive interactions with Pex5p (PDB ID 3R9A; Fodor et al., 2012).

### Future Directions

The reaction mechanism of human AGT1 has been described in detail (Giardina et al., 2017), and because the catalytic residues are conserved in *Arabidopsis* AGT1, this protein likely has the same mechanism. Recent structures of human AGT1 (Giardina et al., 2017) show evidence of X-ray radiation-induced reduction and breakage of the internal aldimine bond between PLP C4′ and Lys209 Nε (equivalent to Lys201 in *Arabidopsis* AGT1), together with a change in tilt of the PLP ring to optimize the hydrogen bond between Asp183 (Asp175 in *Arabidopsis* AGT1) and PLP N1 (**Figure 4**). It is possible that radiation damage also caused the lack of the internal aldimine bond observed in the *Arabidopsis* AGT1 structure since data collection time was on the order of hours at room temperature. The internal aldimine bond also was not observed in the structure of AGT from *Anabaena*, even though data used for this structure were collected using cryo-cooled crystals with a synchrotron (Han et al., 2005). Future structural analyses using shorter exposures with an appropriate radical scavenger in the cryosolvent may reveal whether *Arabidopsis* AGT1 possesses an intact internal aldimine bond with PLP and provide additional data that could enable computational studies of the enzyme mechanism.

## Methods

### Purification Of Recombinant AGT1

Untagged recombinant AGT1 from *Arabidopsis thaliana* (AGI: At2G13360) was expressed in *E. coli* BL21(DE3) cells containing an expression plasmid derived from pET28a (Novagen) as previously described (Liepman and Olsen, 2001). For crystallization experiments, AGT1 was purified using a two-step purification procedure that exploits the affinity of AGT1 for its cofactor PLP (Hondred et al., 1985). PLP was bound to a Q-Sepharose FF (Pharmacia) anion exchange column prior to application of the crude soluble fraction of the cell lysate. AGT1 was eluted from the column using a linear gradient of KCl (0–250 mM) in buffer containing 50 mM Tris-HCl, 0.2 mM PLP, and 10% glycerol (pH 8.5). Peak fractions identified using SDS-PAGE and activity assays (Liepman and Olsen, 2001) were pooled and concentrated using an Amicon stirred ultrafiltration unit with a membrane MWCO of 10,000. The concentrated sample was applied to a Superose 6 gel filtration column (Pharmacia) pre-equilibrated with buffer containing 50 mM Tris-HCl, 0.2 mM PLP, 10% glycerol, and 50 mM NaCl (pH 8.5). Peak gel filtration fractions were pooled and concentrated to 11 mg/ml using an Amicon stirred ultrafiltration cell. **Supplementary Figure 2** shows SDS-PAGE analysis of the purified protein.

### Crystallization of AGT1

AGT1 was screened for crystallization by the microvapor diffusion technique using precipitants from Crystal Screen I (Hampton Research). Crystals with different morphologies appeared after several days in several of the precipitant conditions. After optimization, large, greenish-yellow crystals were grown from drops containing equal volumes of protein (11 mg/ml in 0.2 mM PLP, 10% glycerol, 100 mM Tris-HCl pH 8.5) and precipitant (4.1–4.2 M sodium formate) equilibrated against the same precipitant at room temperature (**Supplementary Figure 2**). Crystals grown in the same buffer containing 20 mM l-serine grew more consistently and were clear. Typical crystals grew to ∼0.5 &times; 0.3 &times; 0.2 mm within a week.

### MIR Phasing and Structure Refinement

Since initial attempts to flash-cool the crystals were unsuccessful, all data sets were collected at 25°C in capillary mounts. One-degree oscillation images (exposure ≈5 min) were collected on a Rigaku/MSC R-AXIS IV detector/rotating anode generator (100 mA, 48 kV). Reflections were indexed, integrated, and scaled with the HKL package (Otwinowski and Minor, 1997). Native AGT1 crystals typically diffracted to at least 2.4 Å resolution, and some up to 2.2 Å. AGT1 crystals were orthorhombic, in space group *P*2_1_2_1_2, and with unit cell parameters *a* = 140.2 Å, *b* = 62.8 Å, *c* = 98.2 Å. Two molecules were present in the asymmetric unit.

Native crystals grown in the presence of PLP but without l-serine were colored and expected to be in the internal aldimine form. Diffraction data statistics for such crystals are shown in **Table 1**. Unmerged reflections were not available for the native data, so CC1/2 values could not be calculated. Data (not shown) were also collected from four heavy-atom derivative crystals: mercury chloride, gold cyanide, potassium tetrachloroplatinate, and *p*-hydroxymercuribenzoate. Data to 2.1 Å resolution were collected from the crystals grown in 20 mM L-serine.

MIR phases were calculated by the *SOLVE* program (version 2.03) (Terwilliger, 2003) with four heavy atom derivative data sets. The final mean figure of merit <*m*> was 0.44 for 16,917 reflections from 20.0 to 3.0 Å. Density modification by *RESOLVE* (Terwilliger, 2003) raised the <*m*> to 0.66 with a mean phase change of 44° from the MIR phases. A model of AGT1 containing ∼84% of the amino acid residues was constructed into the resulting 3.0 Å. This model was transformed to generate the other molecule in the asymmetric unit, and its orientation was optimized by real-space rigid body minimization. The remainder of the structure was built with *Coot* (Emsley et al., 2010) and refined with *CNS* (Brünger et al., 1998) with noncrystallographic symmetry restraints between the two molecules in the asymmetric unit. The structure was further refined without NCS restraints in *Phenix* to 2.2-Å resolution to a *R*_cryst_ of 0.139 (*R*_free_ = 0.170) and contains two AGT1 molecules, two PLPs, one formate, one chloride, and 342 water molecules (**Table 1**). The structure from crystals grown in the presence of l-serine was refined with *Phenix* to 2.1 Å resolution with an *R*_cryst_/*R*_free_ of 0.115/0.166 and contains two AGT1 molecules, two PLPs, one hydroxypyruvate, and 422 water molecules. Coordinate files and structure factors have been deposited in the Protein Data Bank as entries 6PK3 (native AGT1) and 6PK1 (AGT1 grown in presence of 20 mM Ser).

Interface and solvent accessibility calculations were done with the PISA server (https://www.ebi.ac.uk/pdbe/pisa/). All figures were prepared with *PyMOL* version 2.2 (Schrödinger LLC).

## Data Availability Statement

The datasets generated for this study can be found in the Protein Data Bank (www.rcsb.org), Accession IDs: AGT1 native, 6PK3; AGT1 with serine, 6PK1.

## Author Contributions

AL and BH purified AGT1 protein; AL crystallized the protein, AL, JV, DP, and BH collected diffraction data; JV calculated phases; JV and MS refined the structure. AL and MS wrote the manuscript and made the figures; AL, JV, DP, BH, LO, and MS edited the manuscript.

## Conflict of Interest

The authors declare that the research was conducted in the absence of any commercial or financial relationships that could be construed as a potential conflict of interest.
