# Supplementary material for: Crystal Structure Of Photorespiratory Alanine:Glyoxylate Aminotransferase 1 (AGT1) From Arabidopsis thaliana
Source: Front Plant Sci. 2019 Oct 11;10:1229. doi: 10.3389/fpls.2019.01229 (PMC6797613; doi:10.3389/fpls.2019.01229)
Supplement: Supplementary file 1 [file DataSheet_1.pdf]

Supplementary Material

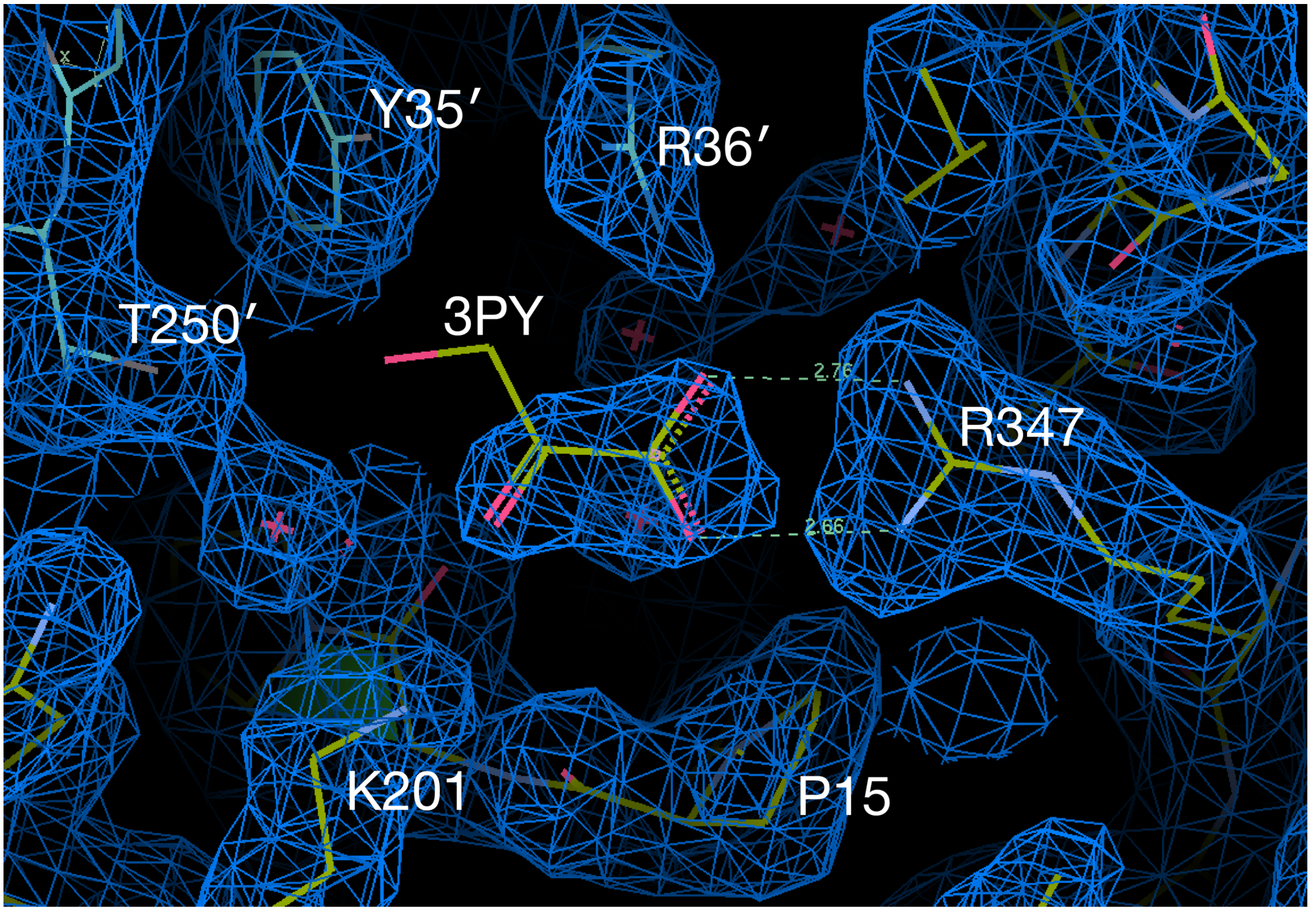

Supplementary Figure 1. Hydroxypyruvate (3PY) refined in the active site of Aradopsis AGT1. Hydroxypyruvate, a catalytic intermediate following transfer of the amino group from serine, was fit into the difference electron density shown in Figure 5A and refined with *Phenix*. Shown is electron density after refinement in the vicinity of the modeled ligand, calculated with coefficient $2mF_{obs}–DF_{calc}$ (blue lines) and contoured at 1.0 times the root-mean-square above the mean of the map, Note that no electron density appears for the hydroxymethyl group.

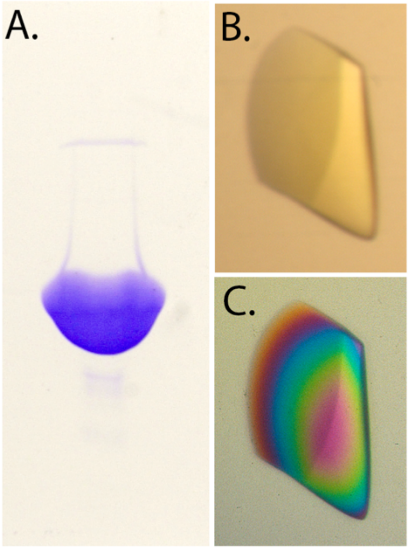

Supplementary Figure 2. (A) A sample of ~55 μg of recombinant Arabidopsis AGT1 purified using anion exchange and gel-filtration chromatography was separated using SDS-PAGE and stained with Coomassie blue. The gel lane was intentionally overloaded to highlight purity of recombinant AGT1. Representative Arabidopsis AGT1 protein crystal photographed using a dissecting microscope with conventional optics (**B**), and through a polarizing filter (**C**).
